# Five Mechanisms of Manipulation by Bacterial Effectors: A Ubiquitous Theme

**DOI:** 10.1371/journal.ppat.1002823

**Published:** 2012-08-23

**Authors:** David M. Anderson, Dara W. Frank

**Affiliations:** 1 Department of Microbiology and Molecular Genetics, Medical College of Wisconsin, Milwaukee, Wisconsin, United States of America; 2 Center for Infectious Disease Research, Medical College of Wisconsin, Milwaukee, Wisconsin, United States of America; University of North Carolina at Chapel Hill, United States of America

Ubiquitin, a highly conserved polypeptide of 76 amino acids, participates in a vast range of eukaryotic cell processes through its role as a reversible post-translational modifier (see review [1], Figure 1A). Such extensive utilization of a single protein within a host cell lends itself to be an ideal target for microbial manipulation. Host-pathogen co-evolution has endowed present-day pathogens with an ever-expanding repertoire of proteins that function to modulate this system. The majority of these proteins are effectors of type III secretion (T3S) or type IV secretion (T4S) pathways, which are major virulence determinants of many Gram-negative pathogens [2], [3]. This review is focused on five distinct mechanisms in which secreted bacterial effector proteins exploit the host ubiquitylation system (Figure 2).

**Figure 1:**
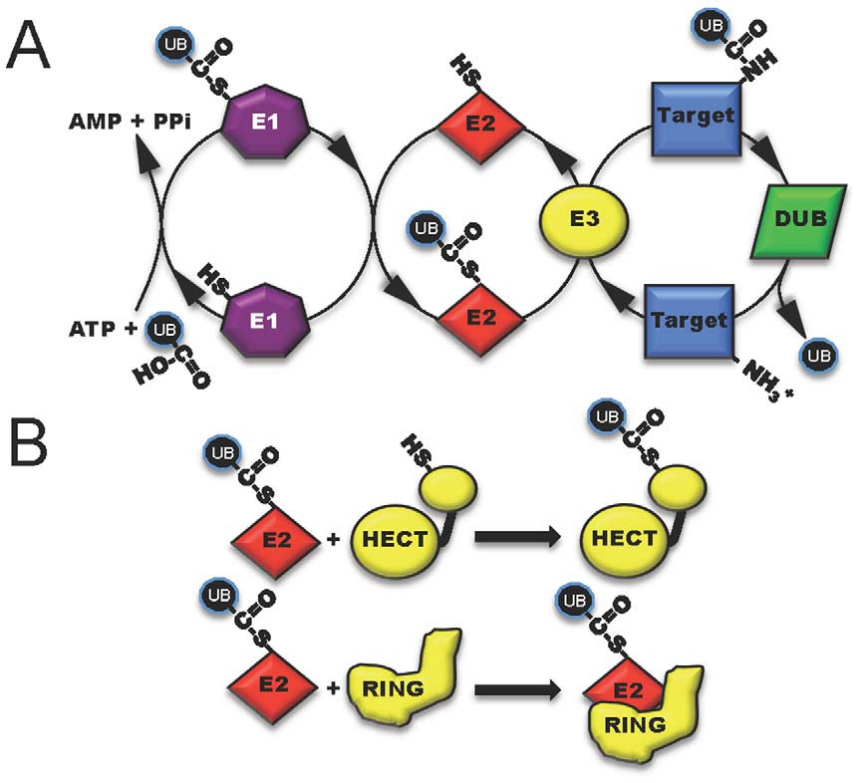
The three classes of host enzymes (E1, purple; E2, red; E3, yellow) involved in ubiquitin modification of target host proteins. (A) E1 (activating enzyme, purple) charges ubiquitin in an ATP-dependent manner to form an E1-ubiquitin thioester intermediate. Activated ubiquitin is then transferred to the conjugating enzyme E2 (red). Target specificity (blue) is determined by E3 ligating enzymes. Linkage to the final modified target protein is by an isopeptide linkage. Deubiquitylase enzymes (DUB, green) can remove ubiquitin for recycling. (B) The two classes of host E3 ligases are illustrated in yellow as HECT and RING. HECT enzymes have a conserved cysteine residue and participate in catalysis. RING enzymes serve as adaptor-like proteins.

**Figure 2:**
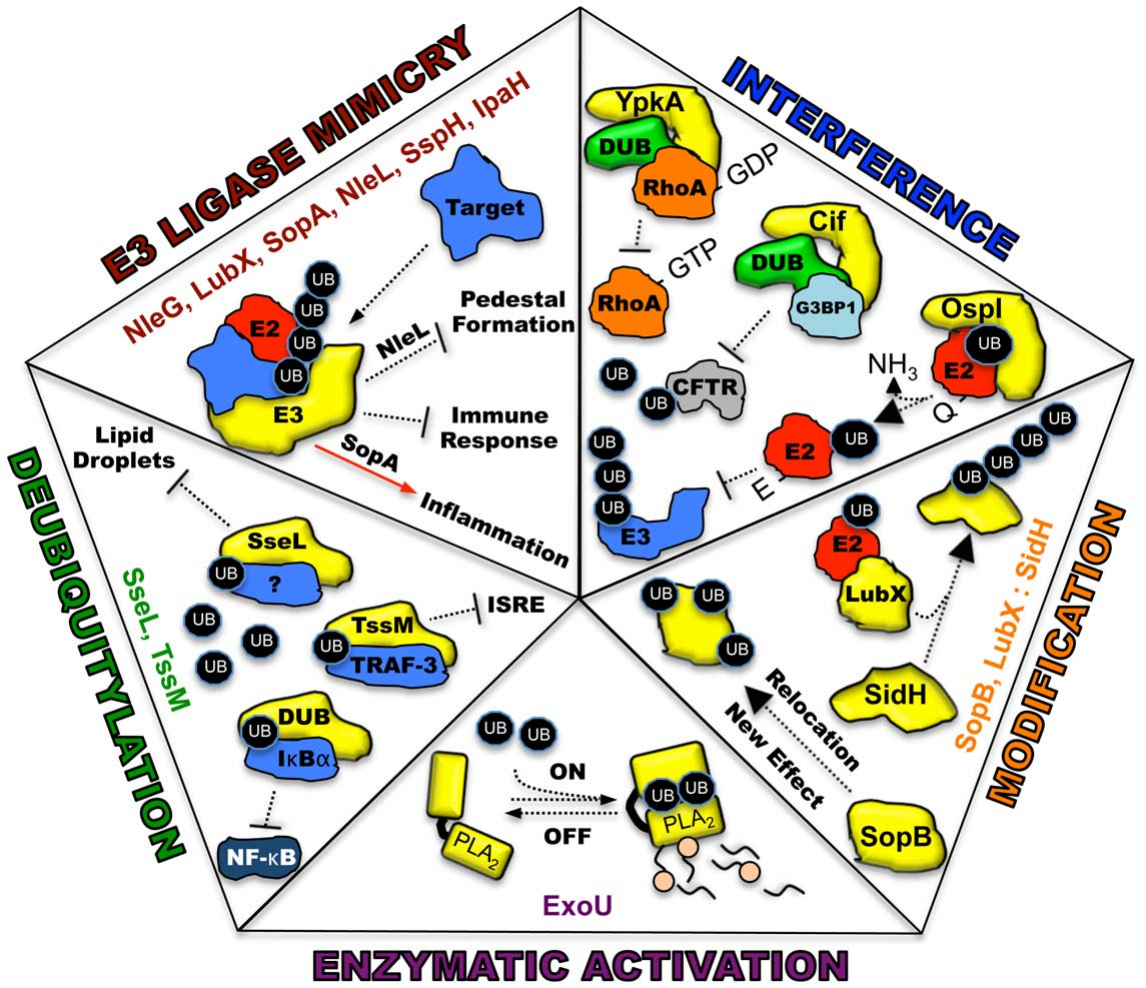
Five distinct mechanisms in which bacterial proteins manipulate the host ubiquitin system. Each piece of the pentagon illustrates a known mechanism for the intersection between pathogen effectors and the host ubiquitin system. Bacterial proteins utilizing each specific mechanism are listed. All bacterial effectors are depicted in yellow. Host target proteins are identified when known and labeled as (?) when unknown. E2, E2 conjugating enzyme, red; E3, E3 ligating enzyme; DUB, deubiquitylating activity, green; UB, monoubiquituin or ubiquitin chains, black ovals; NF-κB, nuclear factor kappa B; IκBα, I kappa B alpha; PLA_2_, phospholipase activity with A_2_ specificity.

## E3 Ubiquitin Ligase Mimicry

One method to disrupt host cell physiology involves the injection of bacterial effectors that mimic proteins in the final step of ubiquitylation involving the E3 ligases [4]. Eukaryotic cells possess two main, functionally distinct classes of E3 ligases: RING-finger (really interesting new gene) and HECT-type (homologous to the E6-associated protein C-terminus) enzymes. RING (and related U-box) ligases act as bridging partners between E2-ubiquitin conjugates and target proteins, while HECT ligases directly participate in the chemistry of ubiquitylation (Figure 1B) [5], [6]. Bacterial effectors mimic both RING and HECT-class ligases and include a newly discovered third mechanistic class, appropriately named novel E3 ligases or NEL [7].

Recently characterized examples of bacterial ligases resembling the RING-finger/U-box enzymes include NleG, encoded by enterohaemorrhagic *Escherichia coli*, and LubX, encoded by *Legionella pneumophila*. The NleG family contains a conserved C-terminal domain that interacts with human E2 enzymes in a similar fashion as their E3 eukaryotic counterparts. The intracellular targets for NleG ligase activity, however, are unknown [8]. LubX is a novel, double U-box-containing enzyme. The first U-box is critical for ubiquitin ligase activity, while the second is necessary for targeting substrates such as the kinase, Clk1 [9]. A recent report revealed an additional target for LubX (see Effector Modification). Bacterial HECT-like ligases such as SopA (*Salmonella*) and NleL (*E. coli* O157:H7) each contain a conserved catalytic cysteine residue and interact with the E2 enzyme, UbcH7, on the same surface as eukaryotic ligases [10], [11]. The structural flexibility of their C-terminal subdomains, which are required for transthiolation of ubiquitin, appears similar between bacterial and eukaryotic enzymes as well [12]. NEL family members are related to HECT E3s because of the formation of a thioester bond with ubiquitin via a conserved cysteine in the catalytic domain. However, they differ in their mechanisms of interacting with substrates. SopA/NleL HECT-like enzymes possess a flexible, bilobed catalytic domain while novel ligases IpaH3 (*Shigella*) and SspH2 (*Salmonella*) likely undergo a dramatic reorientation between their N-terminal leucine-rich repeat and C-terminal NEL domains upon substrate recognition and before catalysis [7], [12]–[14]. Collectively, these mimics are profound examples of convergent evolution and the utilization of different strategies for solving a similar biological problem.

## Deubiquitylation

Many bacteria synthesize effectors that interfere with host ubiquitylation by mimicking host deubiquitylases (DUBs). Eukaryotic DUBs exhibit exquisite substrate specificity and are important regulatory components within the host ubiquitin system [15]. Bacterial DUBs are generally modeled after eukaryotic cysteine proteases and are commonly used to attenuate NF-κB-related inflammatory responses by deubiquitylating and stabilizing IκBα. The *Burkholderia pseudomallei* DUB, TssM, additionally targets lysine 63-linked TNFR-associated factor-3 (TRAF-3) and TRAF-6, affecting interferon stimulated response element (ISRE) signaling and IKK activation, respectively [16]. Other mechanisms include the alteration of the intracellular environment. The DUB activity of *Salmonella* SseL was recently shown to affect lipid metabolism in infected gallbladder epithelial cells to prevent the accumulation of lipid droplets. These results suggest that in addition to IκBα, SseL possesses other target substrates [17]. Such disruption of complex cellular processes by the activity of a single enzyme provides both merit to the vulnerability of the host ubiquitin system to manipulation and insight into eukaryotic cell physiology.

## Effector Modification

Bacterial pathogens have also evolved effectors that allow the host ubiquitin system to fine-tune their function, localization, or temporal regulation. One fascinating example of temporal regulation was revealed for two *Salmonella* Rho GTPase-modulating enzymes, SopE and SptP. SopE, a guanine exchange factor (GEF), is rapidly degraded by the host proteasome via ubiquitylation. SptP plays the opposing role of a GTPase activating protein (GAP) and differs in amino acid sequence resulting in a longer half-life compared to SopE. Thus, bacterial internalization is facilitated through membrane ruffling induced by SopE GTPase stimulation. Membrane and cytoskeletal homeostasis is then achieved though SopE degradation and the opposing actions of the longer-lived SptP [18]. During the intracellular replication of *Legionella pneumophila*, the bacterial E3 ligase mimic, LubX, regulates the stability of a second effector, SidH, via ubiquitylation. In the absence of LubX, SidH is stabilized, which leads to a hyper-virulent phenotype in a *Drosophila* infection model. LubX was thus coined to function as a “metaeffector,” a remarkable testament of pathogen co-evolution with its host [19].

Diversification of effector function and localization through ubiquitylation has been demonstrated for the *Salmonella* phosphoinositide phosphatase, SopB. In this case, an enzyme with a single catalytic function can act to modulate multiple cellular processes depending on its ubiquitylation state. SopB first traffics to the host plasma membrane, affecting actin reorganization and bacterial entry, macropinocytosis, and Akt activation. Post-multimonoubiquitylation by host enzymes, these activities are downregulated and SopB relocalizes to the *Salmonella*-containing vacuole to alter vesicular trafficking for the promotion of bacterial replication [20]. In sum, each of these strategies uses the host ubiquitin system to minimize the genetic cost of maintaining multiple virulence factors by simplifying the number of effectors required to alter host cell function.

## Signaling Interference

There is a growing array of effectors that function to interfere with host ubiquitylation by mechanisms that differ from direct ubiquitylation/deubiquitylation. For instance, the *Shigella flexneri* protein, OspI, inhibits NF-κB signaling through the E3 ligase, TRAF6. Structural and mass spectrometry-based investigations indicate that OspI deamidase activity modifies an E2 enzyme (UBC13) recognizing TRAF6. Deamidation of glutamine 100 to glutamate on the E2 prevents polyubiquitylation of the E3 and activation of downstream innate immune response [21].

Host enzymes such as DUBS can also play scaffolding roles for bacterial effectors to enhance virulence. *Pseudomonas aeruginosa* uses outer membrane vesicles to export a protein known as Cif. Cif interferes with the endosomal recycling of the cystic fibrosis transmembrane conductance regulator (CFTR) to ultimately inhibit chloride secretion. Functionally, Cif stabilizes an intracellular complex of a DUB (USP10) and G3BP1, preventing deubiquitylation of CFTR, which is required for recycling the receptor to the plasma membrane [22]. Lastly, YpkA, a multifunctional serine/threonine kinase of *Yersinia*, has been postulated to sequester a phosphorylated DUB (OTUB1) and GDP-bound RhoA together in a complex. As OTUB1 activity and GTP-RhoA are implicated in enhancing bacterial uptake, inhibition of each may serve a role in preventing bacterial internalization and cell death [23]. The finesse of each of these effectors greatly enhances their specificity and prevents unintended collateral damage that might stimulate further inflammatory responses.

## Enzymatic Activation

Our group has recently discovered a fifth mechanism of pathogen manipulation of host ubiquitylation. *Pseudomonas aeruginosa* possess a suite of T3S effectors known as ExoS, ExoT, ExoU, and ExoY [24]. Each enzyme contains domains that are catalytically inactive until injection into a target cell and association with a specific eukaryotic cofactor. Mechanistically this strategy ensures that each protein can be safely synthesized within *Pseudomonas*. We identified ubiquitin as such a cofactor for ExoU, a patatin-like A_2_ phospholipase with potent cytotoxic activity [25]. This appears to be the first example of a bacterial enzyme specifically requiring ubiquitin for catalysis. Co-expression of ExoU and ubiquitin in a prokaryotic system results in membrane damage and cell lysis, suggesting that no host ubiquitylation components, other than monoubiquitin, are required for phospholipase activity. Analysis of the enzyme activity in vitro indicates that multiple isoforms of ubiquitin activate ExoU, including ubiquitylated proteins and purified polyubiquitin chains with a variety of linkages [26]. Interestingly, a diubiquitin chain modifies ExoU soon after injection, an event with potential trafficking repercussions but not affecting overall toxicity [27], [28]. It is unclear whether the ubiquitylation of ExoU is part of the intracellular activation mechanism. Bioinformatic searches suggest as many as 4,400 bacterial proteins with typical patatin domains reside within sequenced bacterial genomes [29]. As many of these proteins remain uncharacterized, it is tempting to speculate that some of them may also require eukaryotic proteins such as ubiquitin or specific ubiquitylated proteins for activation.

## Conclusion

Ubiquitin is an extremely conserved protein used for an ever-increasing array signaling cascades and regulatory events within all eukaryotic cells. During host-pathogen co-evolution, microbes have exploited these essential pathways to diversify and regulate the function of their effectors. The absence of ubiquitin and its related machinery from prokaryotes makes it a safe and effective target. Continued research into the host-pathogen relationship will no doubt reveal additional mechanisms by which bacterial effectors usurp the host ubiquitin system. Discovery of such mechanisms will provide a basis for therapeutic intervention as well as reveal new aspects of eukaryotic physiology.
